# Burnout and anxiety levels in human medicine teachers, COVID-19 context

**DOI:** 10.12688/f1000research.110498.1

**Published:** 2022-05-04

**Authors:** Jorge Guillermo Morales Ramos, María Susana Picón Pérez, Freddy Albaro Manayay LLaguento, Enaidy Reynosa Navarro

**Affiliations:** 1 Facultad de Medicina Humana, Universidad De San Martín de Porres, Chiclayo, Lambayeque, 14011, Peru; 2 Instituto de Investigación en Ciencia y Tecnología, Universidad César Vallejo, Trujillo, La Libertad, 13001, Peru

**Keywords:** Anxiety; Burnout Syndrome; Distance education; E-learning; COVID-19

## Abstract

**Introduction: **In the COVID-19 context, university teachers have had to face the most complex educational demands, psychosocial risks, and the anxiety of responding to limitations in terms of connectivity and fulfillment of academic objectives **. **To identify the levels of Burnout and anxiety in the COVID-19 context and determine how these levels are manifested in the participating teachers.

**Methods**: This was an analytical non-experimental, cross-sectional study. The population was 150 teachers of the Human Medicine Program of the University of San Martín de Porres, Chiclayo, Peru, and the sample was 66 teachers. The survey consisted of three sections: 1. Informed consent, 2. Maslach's Burnout Inventory, 3. Beck's Anxiety Inventory. Data processing was performed using the SPSS V.27 statistical software and all citations and bibliographical references were processed using Mendeley Desktop 1.19.8.

**Results:** In the variable burnout syndrome, 25% of the participants were in the high level downwards; they present anxiety in 30.30% of the total. It was found that 50% of teachers presented mild to moderate anxiety.

**Conclusions:** the largest number of teachers surveyed present anxiety due to burnout syndrome in the COVID-19 context. Finally, it is found that there is a correlation between anxiety and the sociodemographic variables sex, age, and marital status.

## 1. Introduction

Burnout syndrome (SBO) is defined as a psychological syndrome resulting from certain interrelated experiences, characterized first by emotional exhaustion, as a response to stress; second, by depersonalization, which usually presents as an adverse change in how the person feels about work and other people (cynicism); and third, by low personal fulfillment, which is when people begin to feel negative about themselves, about their ability, their competence, their motivation to work. ^1^^–^^4^ The World Health Organization (WHO) officially recognized this syndrome as a disease associated with work and as an occupational risk factor due to its ability to affect quality of life, mental health and even put lives at risk of the individual who suffer from it; however, this resolution of its incorporation will enter into force in 2023. ^5^ SBO is also known as emotional exhaustion syndrome or burnout syndrome (SQT) and is considered a psychological response of an interpersonal and emotional nature, exclusive to professionals who provide services with high social or help demand. In short, it is one of the most damaging types of stress that arises as a result of chronic work stress, which is influenced by individual, social and organizational variables. ^6^ There are factors associated with Burnout for teachers, including sociodemographic variables (sex, age, marital status, level taught and type of center), personality (external locus of control, higher level of self-awareness, self-efficiency and self-control, behavior pattern, low-level self-esteem), and work and organizational factors, such as work overload. ^7^

Anxiety is defined as a response system at a cognitive, physiological, behavioral, and affective level, which occurs when anticipating circumstances that are usually judged as very aversive when perceived as unexpected events, which are out of control and that can enhance threats to the interests in people's lives. ^8^ Anxiety manifests itself differently in subjects due to biological and learned factors. Some individuals may react with anxiety when faced with threatening situations, while others, in similar situations, will respond by attaching less importance to the facts. ^9^^,^^10^ Most anxiety theory and research now recognize five subtypes of anxiety disorders: these are panic attacks, generalized anxiety disorder, social phobia, obsessive–compulsive disorder (OCD), and post-traumatic stress disorder (PTSD). ^8^ According to WHO, anxiety is one of the factors that contributes to global disability, placing it in sixth position (3.6%), being more common in women than in men (4.6% and 2.6%); in 2015, according to his calculations, almost 264 million people suffered from some type of anxiety disorder. Anxiety is a common mental disorder that involves behaviors or manifestations that include physical, mental, or emotional agitation in the face of specific events or daily life that involve uncertainty and instability. ^11^^,^^12^ Rates can vary, from 2.9% in the Western Pacific region to 5.8% in the Americas region. ^13^

In the coronavirus disease 2019 (COVID-19) context, the population's mental health has been compromised, especially in those groups present on the front lines. Among these groups, we can find the professionals who work as teachers in the Human Medicine Programs (PMH), who, in the conditions of the pandemic, have had to face the demands, the psychosocial risks, the anxiety caused by responding to the limitations in terms of connectivity and fulfillment of academic objectives, all of this in a context marked by academic and technological barriers, conditions of spaces in the home, interruption of work by relatives, among other problematic manifestations. ^14^^–^^16^

### 1.1 Literature review

There is still insufficient research to analyze the impact caused by COVID-19 on people's mental health. A study carried out in China, whose objective was to evaluate the psychological impact of COVID-19 on its inhabitants to understand better their levels of psychological impact, anxiety, depression, and stress during the initial stage of the pandemic—based on 1,210 respondents from 194 cities—found that 53.3% of the participants rated the psychological impact of COVID-19 as moderate or severe, 16.5% reported moderate-to-severe depressive symptoms, 28.8% reported anxiety symptoms moderate to severe and 8.1% reported moderate-to-severe stress levels. ^17^

Also, a systematic review, which included 13 studies, concluded that mental health and mental functions of health professionals were compromised, mainly in those on the front line. The results of this study were as follows: medium-high levels of anxiety (26.5–44.6%), depression (8.1–25%), and worry and insomnia (23.6–38%). ^18^ Another study in university teachers, whose purpose was to identify the prevalence and factors associated with SBO, found worrying levels of Burnout; the most frequent associated factors were: accumulation of extra-teaching activities, work overload, high labor ties, low remuneration, and devaluation of the work performed. ^19^

A cross-sectional investigation that included 459 teachers (61% women); found a 20.8% prevalence of SBO, 10.6% for the rate of depression, and, regarding anxiety, the rate was 30.9%. Likewise, the comorbidity among the participants was 19.7%. The teachers with SBO presented increased depression and anxiety, significantly associating emotional exhaustion with anxiety and depression more than with depersonalization or personal fulfillment. ^20^ Another study in Spanish university teachers (621 university teachers), whose objective was to evaluate the psychosocial risks of university teachers and identify areas for improvement for a healthy organization; found that these teachers presented an unfavorable situation for their health in five psychosocial dimensions: low self-esteem, high job insecurity, high psychological demands, high double presence and low social support. ^21^ Likewise, an investigation focused on determining Burnout in teaching workers of the Faculty of Nursing of the Cooperative University of Colombia (sample of 30 teachers, population, 56); found that 66.6% presented low emotional exhaustion; 26.6%, average emotional exhaustion; 96%, low level of depersonalization; and 80%, higher level of personal fulfillment. ^22^

1.1.1 Burnout and anxiety: COVID-19 context

Pressley's research (2021), focused on obtaining information regarding the impact of COVID-19 and its association with anxiety in teachers, found that the closest significant predictors to Burnout according to the COVID-19 Anxiety Scale (CAS). These were: anxiety-related teacher burnout, anxiety when communicating with parents, current anxiety about teaching, and administrative support. The research points out that, to limit teacher burnout, it is necessary to monitor them during the pandemic and provide them with permanent educational, technological or emotional support. In addition, educational institutions must provide clear communication and protocols to help educators feel safe amid this complex pandemic. ^23^

Another study in Canadian teachers where the authors investigate whether teachers' attitudes toward change, teaching effectiveness, and attitudes toward technology are correlated with resilience and Burnout during the early stages of a pandemic; also whether perceptions of principal support, teaching effectiveness, attitudes towards ICTs, teacher resilience and Burnout have changed during the early stages of a pandemic; found that attitudes towards change and administrative support were positively correlated with teachers' Burnout and resilience at the start of the pandemic, and that, during the three months following the start of the pandemic, teachers showed an increasing increase in tiredness and cynicism, but at the same time greater efficiency for classroom management and a greater sense of achievement; attitudes, on the contrary, became more negative. On the other hand, the implications of the scarcity of resources were evidenced, which resulted in stress and exhaustion over time. Research suggests that teachers have been burned out by these disruptive changes. ^24^

A recent study in Moldova to evaluate the Burnout of teaching staff during COVID-19 revealed significant relationships between Burnout and some sociodemographic variables such as age, gender, and years of professional experience, showing that female teachers presented higher exhaustion less personal fulfillment than teachers. The temporary closure of educational institutions and the transition to virtual teaching brought some restrictions that produced sudden changes in the teaching-educational environment and, consequently, negative consequences and an impact on teachers' health. The study warns about a high level of Burnout due to these changes in teaching methods. The fundamental concerns of the teachers were located in aspects such as the quality of the lesson, which depends on the teacher being competent to achieve quality electronic learning in the student, beyond the consequences that this means for their comprehensive health. ^25^

For their part, Pressley and Ha (2021) explored the new approaches and requirements of virtual teaching and their impact on the effectiveness and participation of teachers in the USA, based on the variables participation and instruction, taking into account the Scale of Teacher Effectiveness (TSE). The results showed that the mean efficacy scores on the instruction and participation variables were lower than the TSE scores of instruction and participation compared to previous studies. The study also indicates that the teachers of the virtual modality presented lower scores than those who taught following a blended or face-to-face model. The regional educational administration should have provided more support and instructional guidance. ^26^ Using this same scale, another study evaluated changes in Burnout and its relationships with changes in ERT during COVID-19, applying a regression model of latent change in German teachers, namely the implementation of the learning materials during the pandemic it became a relevant process where the evaluation of attitudes and self-efficacy towards virtual learning was taken into account and whether the two variables were related to changes in Burnout and self-efficacy. This study showed that the components of Burnout, depersonalization, and lack of personal fulfillment increased significantly from pre to post COVID-19, while emotional exhaustion did not show significance. Regarding changes in Burnout, the study itself showed a negative correlation with changes in the TSE and established that the lack of resources had an important implication. ^27^

Finally, an investigation in the USA, through an online survey involving 47 volunteer teachers from 18 countries whose objective was to analyze the problems of teacher burnout in general during the pandemic; found that the average level of pre-pandemic Burnout reached 3.8 points (out of a maximum of 10) and during the pandemic, it was calculated at 5.5 (out of a maximum of 10). In addition, the highest level of pre-pandemic Burnout was found in the group with 11 to 20 years of experience, while the level of pandemic burnout was recorded in the group with 21 to 30 years of experience. Among the most relevant characteristics of Burnout found were the psychological ones (stress, discouragement, depression, exhaustion, guilt, frustration, panic, and tiredness), those that were related to health (weakness, headache, insomnia, spine, and eyes), educational (lack of resources, increased workload, face-to-face contact, multitasking, cheating and low motivation of students), technological (spending too much time on the computer and the quality of the internet), management (lack of student participation, insufficient reward system, and excessive administrative control), health (excessive administrative control and lack of students), lifestyle, family and financial problems. ^28^

The research results consulted so far show worrying rates of Burnout associated with anxiety in teachers globally. This reality is not unrelated to the situation that the professors of the Human Medicine Program of the University of San Martín de Porres, Chiclayo, Peru (PMH-USMP-Chiclayo, Peru; by its acronym in Spanish), have been facing; who, since the beginning of COVID-19, have been facing multidimensional difficulties. In the personal aspect, they faced insecurity to face the new educational forms that included using technological tools hitherto unknown or little-used; according to the results obtained, this situation generated high anxiety levels in the participants. In the labor aspect, the main limitations were work overload, the lack of adaptation to new schedules, the lack of economic resources, teaching materials, modern technological equipment, and connectivity problems. In the academic aspect, the lack of training in the management of educational technology, lack of experience in virtual teaching, deficiencies in the development of evaluation processes in the student, deficiencies in the feedback of content with the student as a result of the technological barriers between teacher-student-parents, deficiencies in the management of new educational methodologies for virtual teaching.

All these manifestations were decisive to problematize: what are the levels of Burnout and anxiety in the COVID-19 context in teachers of the Human Medicine Program of the USMP, Chiclayo, Peru? The objective of this research was to identify the levels of Burnout and anxiety in the COVID-19 context; also determine how these levels are manifested in the teachers participating in the study. As a hypothesis, it is argued that the Burnout Syndrome increases the anxiety of the teaching staff of the PMH-USMP-Chiclayo, Peru.

## 2. Methods

### 2.1 Study design

The study uses an analytical, non-experimental, and cross-sectional design to respond to a positivist paradigm. A diagnostic evaluation was carried out based on online survey studies, using Google Forms, to measure the effect of COVID-19 on Burnout and anxiety in teachers from the PMH-USMP-Chiclayo, Peru; made up of two academic departments: 1. Basic Sciences and 2. Clinical Sciences; analyzing the variables in their natural context, without manipulating them. SBO was defined as study criteria variables and sociodemographic variables as predictor variables. The SBO variable was classified into three dimensions: emotional exhaustion (nine questions), depersonalization (five questions), and personal accomplishment (eight questions), for a total of 22 items with a Likert-type response scale with seven levels (never, a few times a year or less, once a month or less, a few times a month or less, once a week, a few times a week, and every day). While the anxiety variable was made up of 21 items with a Likert-type response scale with four levels: normal (0–7), mild, ^8^^–^^15^ moderate, ^16^^–^^25^ and severe (26–63); taking into account that there will be anxiety when the levels are mild, moderate or severe. The Chi-squared test was applied to correlate the variables Burnout Syndrome and anxiety; These data are shown in Table 6, which allowed us to establish the null (H0) and alternative (H1) hypotheses.

• H1: The Burnout Syndrome increases the anxiety of the teaching staff of the PMH-USMP-Chiclayo, Peru, in the COVID-19 context.
• H0: The Burnout Syndrome decreases the anxiety of the teaching staff of the PMH-USMP-Chiclayo, Peru, in the COVID-19 context.

### 2.2 Study population

The population was composed of 150 teachers. The sample was non-probabilistic for convenience, consisting of 66 teachers from the PMH-USMP-Chiclayo, Peru. To obtain the sample size, the formula was used to estimate proportions of a known population, considering a Cronbach's alpha of 5% and an allowed error of 9%; obtaining a sample value of 66.4. Inclusion criteria: practicing teachers (full-time dedication, part-time dedication) who have taught uninterruptedly from 2018 to the present; they are registered in the National Superintendence of Higher University Education; and belong to the teaching staff of the PMH-USMP-Chiclayo, Peru. Exclusion criteria: teachers who do not meet the above criteria or whose teaching work has been intermittent from 2018. The distribution of teachers according to sociodemographic variables of age, sex, marital status behaved as follows: age range, 26–32 years (1.52%), 33–40 years (22.73%), 41–50 years (30.30%), and 61–74 years (15.15%). Gender: 76% men and 24% women. Marital status, divorced (1.52%), widowed (4.55%), married (80.30%), and single (13.64%).

### 2.3 Data collection

Data collection took place from March 3, 2021 to April 5, 2021. The data collection technique was the survey, through two questionnaires. The first was the Maslach Burnout Inventory (MBI), which evaluates the manifestation of not being able to give more of oneself, both physically and mentally (exhaustion), the presence or absence of a negative attitude of devaluation and loss of interest in work (depersonalization), and the existence of doubts about one's ability to do academic work (lack of personal accomplishment). ^4^^,^^29^ The second was the Beck Anxiety Inventory, which effectively measures the degree of anxiety in children and adults. ^30^^,^^31^

### 2.4 Data analysis

First, the data was processed, eliminating all the subjects with missing values in all the variables and with scores out of range; second, the total score of the variables SBO (in its three dimensions) and anxiety was obtained, with which the level of the scales for each variable was obtained (adhering to the recommendations of the authors of the instruments); and, finally, the descriptive and inferential data analysis was carried out, through the statistical software SPSS V.27. To limit possible bias in data processing, parallel processing of the same data was used, but with other specialists from the statistics area of the institution that supports this study. In this sense, no significant differences were found.

### 2.5 Ethical considerations

The research complies with the ethical principles proposed in the Declaration of Helsinki. The principles of autonomy and informed consent are considered fundamental elements within the scientific research process. ^32^ All teachers freely participated in the research. They were informed promptly through a meeting *via* Zoom, notified through institutional email, and explained the investigation's purpose. In the end, all signed an informed consent letter where the limits of their participation were established. The Ethics Committee approved the research of the Universidad De San Martín de Porres, Chiclayo, Peru. Date: February 22, 2021, Official Letter No. 139-2021 - CIEI-FMH-USMP. ^33^

## 3. Results

Table 1 shows that in the burnout syndrome variable, 50% of teachers from the PMH-USMP-Chiclayo, Peru, are in the middle level downwards, the same thing happening with the third quartile. However, we have that the last quartile is in high levels downwards. In the anxiety variable, 50% of teachers from the PMH-USMP-Chiclayo, Peru, were normal, indicating that they do not have anxiety. In addition, the third quartile of teachers was at the mild level, which also indicates that they do not have anxiety. However, the last quartile of teachers was at levels less than or equal to 4 (severe downwards) of anxiety.

**Table 1.** Descriptive statistics of the variables.

	Median	p25	p50	p75	p100
Burnout syndrome	2	2	2	2	3
Anxiety	1	1	1	2	4

### 3.1 Burnout syndrome

In Table 2, 57.58% of the teachers are in the medium level of SBO due to emotional exhaustion, and 13.64% are at the high level. Regarding the depersonalization dimension, 46.97% of teachers are at the medium level of SBO and 13.64% at the high level. Regarding the personal achievement dimension, 80.30% of teachers are at the medium level, while 4.55% are at the high level.

**Table 2.** Burnout Syndrome according to dimensions, in teachers of the PMH-USMP-Chiclayo, Peru.

Level	Dimensions		
	Emotional exhaustion	Depersonalization	Personal fulfillment
Low	28.79%	39.39%	15.15%
Medium	57.58%	46.97%	80.30%
High	13.64%	13.64%	4.55%

In Table 3, gender indicator, it can be seen that the largest number of teachers who belong to the male sex, who present Burnout syndrome, have a medium level in emotional exhaustion, ^28^ low level, ^22^ and medium ^21^ in emotional exhaustion. Depersonalization and medium ^42^ in personal fulfillment; in the same way, in the case of females, the medium level predominates ^10^: in emotional exhaustion, ^10^ in depersonalization, and ^11^ in personal fulfillment. In the Age indicator, the largest number of teachers by age, who present burn syndrome, have a medium level: in emotional exhaustion in the age ranges: 33-40, ^9^ 41-50, ^13^ and 51-60 ^10^; in depersonalization in the age ranges: 33-40, ^7^ 41-50 ^12^ and 51-60 ^9^; and, in personal fulfillment in the age ranges: 33-40, ^13^ 41-50, ^17^ 51-60 ^13^ and 61-74. ^9^ Finally, in the Marital Status indicator, it is seen that the largest number of teachers, by marital status, who present burnout syndrome, are married and have a medium level: in emotional exhaustion, ^34^ in depersonalization, ^25^ and personal fulfillment. ^44^

**Table 3.** Burnout compared with the sociodemographic variables of teachers from the PMH-USMP-Chiclayo, Peru.

Burnout dimension	Level	Sex		Age (years)					Civil status			
		F	M	25-32	33-40	41-50	51-60	61-74	S	Ma	W	D
Emotional exhaustion	Low	4	15	1	3	5	7	3	4	12	2	1
	Medium	10	28	0	9	13	10	6	3	34	1	0
	High	2	7	0	3	2	3	1	2	7	0	0
Depersonalization	Low	4	22	1	6	5	8	6	4	21	1	0
	Medium	10	21	0	7	12	9	3	4	25	1	1
	High	2	7	0	2	3	3	1	1	7	1	0
Personal fulfillment	Low	3	7	0	2	2	5	1	1	7	1	1
	Medium	11	42	1	13	17	13	9	8	44	1	0
	High	2	1	0	0	1	2	0	0	2	1	0

* M=Male; F=female; S=single; Ma=married; W=widowed; D=divorced.

### 3.2 Anxiety

In Table 4, anxiety was observed at mild (15.50%), moderate (12.12%), and severe (3.03%) levels, which indicates that teachers presented anxiety in 30.30% of the total. It can also be seen that 69.70% of teachers did not present anxiety.

**Table 4.** Anxiety at its different levels.

Levels	Anxiety
Normal	69.70%
Mild	15.15%
Moderate	12.12%
Severe	3.03%

In Table 5, the largest number of teachers belong to the male gender (75.75%). Anxiety is observed in male teachers: 5 (7.58%) are at the mild level; 8 (12.12%), at a moderate level; and 2 (3.03), at the severe level. This indicates that, of the 16 female teachers, only 5 (7.58%) present anxiety at the mild level. In addition, the largest number of teachers belong to the age ranges between 33 to 50 years, with the following distribution: 7.58% in the light level; in the moderate, 9.09%; and 1.52% in severe. For teachers in the range of 51 to 60 years: 7.58% are included in the light level; at the moderate level, 1.52%; and at the severe level, 1.52%. Concerning teachers in the range of 61 to 74 years, 1.52% are at the moderate level. On the other hand, it must be added that teachers whose age range is between 25 and 32 years do not present anxiety ( Table 3).

**Table 5.** Variable anxiety compared with the sociodemographic variables of teachers of the PMH-USMP-Chiclayo, Peru.

Anxiety levels	Gender		Age (years)				Civil status			
	F	M	25-32	33-50	51-60	61-74	S	Ma	W	D
Normal	16.66%	53.03%	1.52%	34.80%	19.7%	13.64%	12.12%	53.03%	3.03%	1.52%
Mild	7.58%	7.58%	-	7.58%	7.58%	-	1.52%	13.64%	-	-
Moderate	-	12.12%	-	9.09%	1.52%	1.52%	-	10.60%	1.52%	-
Severe	-	3.03%	-	1.52%	1.52%	-	-	3.03%	-	-

* M=Male; F=female; S=single; Ma=married; W=widower; D=divorced.

**Table 6.** Correlation between Burnout Syndrome and Anxiety in teachers of the PMH-USMP-Chiclayo, Peru.

	Value	df	Asymptotic significance (bilateral)
Pearson Chi-squared	13,374 ^a^	6	0.037
N valid cases	66		

Regarding marital status, the following is noted: 83.30% of teachers belong to married marital status, of which 13.64% of them are in the mild level, 10.60 in the moderate level, and 3.03% in the severe level. In addition, mild anxiety is observed in 1.52% of teachers with single marital status. In the widowed, marital status, only 1.52% are moderate ( Table 3).

## 4. Discussion

Based on the results found, the research hypothesis is confirmed given that the most significant number of teachers surveyed from the PMH-USMP-Chiclayo, Peru, present anxiety due to burnout syndrome (medium and high levels) in the COVID-19 context. Also, the null hypothesis is rejected.

### 4.1 Burnout syndrome

Below the median is 50% of participating teachers. A similar situation can be seen in the third quartile. However, the last quartile is at a high level downwards ( Table 1). Various studies found high prevalence rates of burnout: 67.5%, ^34^ 42.1%, ^35^ 20.8%, ^20^ and 40.0%. ^7^ It is necessary to emphasize that the most recurrent causes such as becoming too internalized in the problems of the students, the disproportionate preparation of academic and administrative documentation, work overload, low salary compensation, poor working conditions, and poor recognition of the effort of the teacher, ^35^ must be taken into account to prevent Burnout from affecting not only teachers but also their teaching performance. This study rescues other characteristics: psychological (stress, discouragement, depression, exhaustion, guilt, frustration, panic and tiredness), health (weakness, headache, insomnia, spine and eyes), educational (lack of resources, increased workload, face-to-face contact, multitasking, cheating, and low student motivation), technological (spending too much time on the computer and internet quality), and managerial (lack of student engagement, insufficient reward system and excess administrative control). ^28^ It is also inferred that the rate of teacher burnout is increasing because current educational policies are not aligned with teachers' expectations, in the same way, teacher resilience is depleted due to decreased self-efficacy, the quality of professional relationships and an increase in the pace of technological integration.

High values of 57.58% and average 13.64% were obtained for the emotional exhaustion dimension. In agreement, a consulting study found levels of Burnout of 66.66%. ^22^ These high levels of Burnout are associated with a high commitment at work, work stress, youth and inexperience, academic work, research activities, teaching experience, and teachers' employment status. ^36^^,^^37^ These results allow us to agree that online classes—already in the COVID-19 context— contributed to the social isolation of the teacher caused them emotional exhaustion because they had to develop and comply with new teaching activities to which they were not accustomed, meaning the search for greater motivations and efficient, but stressful, time management. ^38^ In the depersonalization dimension, the teachers presented high values of 13.64%) and average values of 46.97%, showing signs of indifference and cynicism towards the students. These results are corroborated by current research that found burnout levels of 35%, 37.7% and 80% respectively. ^34^^,^^35^^,^^39^ These values help explain why the depersonalization dimension increased significantly in the COVID-19 context compared to the pre-COVID-19 stage.

The personal accomplishment dimension was located at the medium level in a higher proportion concerning the other two dimensions (80.3%) and, at the high level in a lower proportion (4.55%), which are contrasted with the results presented by Araoz & Ramos (2020): 32.8% at the medium level and 27.6% at the high level, ^35^ and those achieved in a study carried out on medical personnel in which they obtained values of 87.1% high level. ^40^ These results show a weak work organization, a weak work structure, as well as a low capacity to face teaching work in the COVID-19 context ^41^; however, it is necessary to understand that the changes from learning in the traditional classroom to the virtual one; meant an enormous challenge for teachers, not having the technological infrastructure, not having had enough time to achieve the appropriate skills for remote teaching or emergency distance education, could have a balance with overwhelming results and frustration. ^42^ A recent study argued that COVID-19 demanded huge sacrifices from teachers, who in record time went from traditional face-to-face education to a new educational modality that directly impacted their self-efficacy. ^26^ It is confirmed that teacher self-efficacy influences a high level of confidence in their skills and abilities for the development of teacher education.

When analyzing the sociodemographic variables according to age, the data were distributed at the average level in all age ranges in terms of emotional exhaustion, depersonalization, and personal accomplishment. It should be noted that in the research by Araoz & Ramos (2020), it was explained that younger teachers have to deal with instability and work overload ^35^; also, Aveiro-Róbalo et al. (2021) argue that at a younger age there is a greater perception in terms of repercussions that are associated with anxiety that translate into exhaustion, fear, worries, and perception of abuse. ^43^ Another study consulted found that the level of Burnout marked a reduction with increasing age in men and women. The bimodal association in women between 20 and 35 and older than 55 showed the highest level of Burnout. ^44^ Regarding the marital status of teachers, there is greater personal fulfillment at the average level in married teachers than single, divorced, and widowed; This is because married people would have acquired strategies to deal with problems and thus feel better about themselves. ^36^^,^^45^

### 4.2 Anxiety

Regarding the anxiety variable, 30.3% of the participants presented anxiety, which corroborates the data found for anxiety in educators of 30.9%, ^20^ and 42.7% in the medical staff. ^44^ Likewise, 50% of teachers presented anxiety of a mild-to-moderate level, which coincides with the data obtained in a recent study carried out on university teachers at the Ibero-American level in the pandemic scenario, where it was observed that the level of anxiety in the majority of them was medium/low, which was associated with a negative institutional perception of teachers. ^14^ Another investigation analyzed the impact of the COVID-19 outbreak on the mental health of health sector professionals, detecting medium-high levels of anxiety of 26.5–44.6%. ^18^ Other factors also contribute to anxiety, such as lifestyle in urban areas, ^46^ and the confinement that is presented as another significant factor. ^47^ The long period of quarantine that the Peruvian population went through may have increased the possibility of acquiring mental and psychological problems, ^43^^,^^48^ like anxiety (16%). ^49^ Other research indicated that anxiety symptoms increased by 28% in the general population. ^50^

Regarding age, the teachers who showed higher anxiety levels were those in the range of 33–50 years, with 18.19%, of which 9.09% presented moderate level anxiety. This result is consistent with two investigations indicating that the age group that presented the highest level of anxiety in the pandemic was those under 30 years of age. ^43^^,^^51^ A factor that would contribute to psychosocial risk is the years of professional experience since younger teachers with little work experience have not obtained the sufficiency to handle the job, ^52^ especially in the age group of 21–30 years of experience. ^28^ The study of Ozamiz-Etxebarria *et al.* (2020) maintains that symptoms were low. The younger age group and those with chronic diseases reported higher symptoms than the rest of the population. ^53^ It should be taken into consideration that the results of this research are based on a population where the majority of teachers from the PMH-USMP-Chiclayo, Peru, are doctors and that, due to the characteristics of the pandemic, those who were in the front line of care were those included in the range between 25–50 years, who performed care work.

Regarding marital status, married teachers exhibit a moderate-to-severe level; however, various studies warn that there is no relationship between anxiety and marital status. ^54^ However, COVID-19 forced governments to implement distance education through the virtual modality, which could generate feelings of dissatisfaction and loneliness in the participants, generating depression due to the increase in family demands vs. labor demands; the latter with the aggravating circumstance of not having enough technological tools to carry out educational work. ^55^

Finally, the research hypothesis is confirmed given that the largest number of participating teachers present anxiety due to burnout syndrome (medium and high levels) in the COVID-19 context. Therefore, according to the anxiety scale, there is a significant direct relationship between burnout syndrome and its closest predictors.

## 5. Conclusions

From the analysis and interpretation of the data related to Burnout in teachers of the PMH-USMP-Chiclayo, Peru, it is concluded that the most recurrent causes that caused Burnout and therefore affect the performance of the participating teachers are based on the level of involvement of these with the problems of the students, the elaboration of the academic and administrative documentation in a disproportionate way, the work overload, the low salary compensations, the bad working conditions, as well as the deficient recognition of the effort. In addition, other psychological causes (stress, discouragement, depression, exhaustion, guilt, frustration, panic, and fatigue), health (weakness, headache, insomnia, spine, and eyes), education (lack of resources, increased workload, face-to-face contact, multitasking, cheating, and low student motivation), technological (spending too much time on the computer and internet quality), and managerial (lack of engagement of students, insufficient reward system and excessive administrative control).

Beyond work stress, inexperience in disruptive change, mandatory compliance with academic work, research work, social isolation, and the aforementioned emotional exhaustion, teachers have shown commitment to their role, assuming the fulfillment of their new teaching activities to which they were not accustomed. This meant looking for new motivations through personal self-management, which meant an enormous challenge for them as they did not have the technological infrastructure and had enough time to achieve the appropriate skills for virtual teaching.

Participating teachers showed anxiety at medium and high levels due to confinement and negative institutional perception. These anxiety levels were associated with the negative institutional perception of the teachers, who did not have the technological equipment and the necessary training to face the new educational challenge from the beginning. Likewise, other factors prevailed with the alterations in their lifestyles and compulsory confinement, which increased the possibility of being exposed to psychological problems that meant a biopsychosocial risk that influenced teaching, especially in younger teachers with little work experience.

### Limitations

The results of this research were obtained during the COVID-19 context in Chiclayo, Peru. These results could vary as time progresses and this pandemic situation is overcome. On the other hand, as teacher burnout is increasing because current educational policies are not aligned with their expectations, there is a depletion of their coping reserves, self-efficacy, and professional and family relationships that must be addressed in successive studies.

## Data availability

### Underlying data

Zenodo: Burnout and anxiety levels in Human Medicine teachers, COVID-19 context https://doi.org/10.5281/zenodo.6326548 ^56^

The project contains the following underlying data:

➢ Statistical Results.xlsx
➢ Processed data.pdf

Extended dataZenodo: Burnout and anxiety levels in Human Medicine teachers, COVID-19 context https://doi.org/10.5281/zenodo.6326548 ^56^

➢ Burnout Syndrome Questionnaire
➢ Anxiety Instrument
➢ Validation by Expert Judgment

Data are available under the terms of the Creative Commons Attribution 4.0 International license (CC-BY 4.0).

**Contribution of the authors** (taking as reference CRediT – Contributor Roles Taxonomy) https://casrai.org/credit/

**Jorge Guillermo Morales Ramos:** Conceptualization; Data curation; Formal analysis; Funding acquisition; Investigation; Methodology; Project administration; Supervision; Validation; Writing – original draft; Writing – review & editing.

**María Susana Picón Pérez:** Conceptualization; Formal analysis; Funding acquisition; Investigation; Writing – review & editing.

**Freddy Albaro Manayay LLaguento:** Data curation; Formal analysis; Investigation; Validation; Writing – review & editing.

**Enaidy Reynosa Navarro:** Conceptualization; Data curation; Formal analysis; Investigation; Methodology; Supervision; Writing – original draft; Writing – review & editing.
