# Supplementary material for: Burnout and anxiety levels in human medicine teachers, COVID-19 context
Source: F1000Res. 2022 May 4;11:491. [Version 1] doi: 10.12688/f1000research.110498.1 (PMC9837455; doi:10.12688/f1000research.110498.1)
Supplement: Peer review history

# Peer review: Reviewer response for version 1

**DOI:** 10.5256/f1000research.122114.r136730

**Published:** 2023-01-12

**Authors:** Víctor Eduardo Horna Calderón

**Affiliations:** 1 Universidad San Ignacio de Loyola, Lima, Peru

This manuscript reveals how university teachers have faced complex educational demands that have caused high levels of Burnout and anxiety in the context of COVID-19; therefore, the current researchers set out to determine how these levels are manifested in the teachers under study. To do this, they proposed a non-experimental cross-sectional analytical study with teachers from the Human Medicine Program at the University of San Martín de Porres, Chiclayo, Peru; finding that, regarding Burnout, 25% of the participants were at a high level downwards; they present anxiety in 30.30% of the total. Likewise, 50% of teachers presented mild to moderate anxiety.

 From a theoretical point of view, the manuscript defines and bases the study variables in the context of COVID-19 based on reliable academic information extracted from databases such as Scopus, Web of Science, and Springer, among others, citing authors with results and recognized trajectory in the area. Methodologically, the study design and population are accurately described. Likewise, it describes how the processing and analysis of the research data were carried out, as well as the ethical considerations that governed said research.

 The researchers concisely describe the results, discuss them based on theories related to the subject, and identify the scientific background and results found during the process, which allows reaching conclusions that support the results, contributions, and the main gaps and limitations found, which allows opening new horizons of study.

Is the work clearly and accurately presented and does it cite the current literature?

Yes

If applicable, is the statistical analysis and its interpretation appropriate?

Yes

Are all the source data underlying the results available to ensure full reproducibility?

Yes

Is the study design appropriate and is the work technically sound?

Yes

Are the conclusions drawn adequately supported by the results?

Yes

Are sufficient details of methods and analysis provided to allow replication by others?

Yes

Reviewer Expertise:

Psicología de la educación (Psychology of Education), Educación (Education), Burnout

I confirm that I have read this submission and believe that I have an appropriate level of expertise to confirm that it is of an acceptable scientific standard.

# Peer review: Reviewer response for version 1

**DOI:** 10.5256/f1000research.122114.r149090

**Published:** 2022-12-19

**Authors:** Juan Luis Rodríguez Vega

**Affiliations:** 1 Universidad Nacional Mayor de San Marcos, Lima, Peru

This research collects the knowledge of how university teachers have assumed specific educational challenges that, at the same time, have caused high levels of Burnout and anxiety during the COVID-19 pandemic context. To achieve this purpose, they presented a non-experimental study with teachers from the medical school of a private university in the north of Peru. The study finds that the participants showed a high level of 25% cases of burnout and 50% presented mild or moderate anxiety.

 Theoretically, the study is based on current research through which it is possible to precisely define the variables, dimensions, and indicators that intervene in the same COVID-19 context; in addition to the methodological precision that the study presents, it may be replicable in other mental health contexts.

 The results of this research are relevant to the international scientific community, especially in the area of ​​public health and intervention.

 Among the strengths of this study, the causes that cause Burnout in teachers and that affect their general professional performance are identified; among them: the excessive level of involvement of the teacher with the students, the disproportion of work generated by the academic part and the administrative functions that lead to work overload in the teacher. In addition, the need for more technological equipment and teacher training to adequately handle the complex educational scenario that COVID-19 has meant. These findings are fundamental so that, currently and looking to the future, educational institutions promote comprehensive policies that allow the complete professional development of teachers, the fulfillment of their essential functions, and that avoid the depletion of coping, self-efficacy, and teacher professional relations.

Is the work clearly and accurately presented and does it cite the current literature?

Yes

If applicable, is the statistical analysis and its interpretation appropriate?

Yes

Are all the source data underlying the results available to ensure full reproducibility?

Yes

Is the study design appropriate and is the work technically sound?

Yes

Are the conclusions drawn adequately supported by the results?

Yes

Are sufficient details of methods and analysis provided to allow replication by others?

Yes

Reviewer Expertise:

Education and Comprehensive Health of the teacher

I confirm that I have read this submission and believe that I have an appropriate level of expertise to confirm that it is of an acceptable scientific standard.
